# Individualized Accelerated Repetitive Transcranial Magnetic Stimulation Targeting the Inferior Frontal Cortex: A Therapeutic Approach for Refractory Obsessive-Compulsive Disorder

**DOI:** 10.1192/j.eurpsy.2026.10644

**Published:** 2026-07-31

**Authors:** R. Chen, Z. Dong, C. Wu, S. Xiang, W. Liu, V. Voon, Y.-J. Zhao

**Affiliations:** 1 Clinical Research Center for Mental Disorders, Shanghai Pudong New Area Mental Health Center, School of Medicine, Tongji University; 2 Department of Psychology, Shanghai Normal University; 3 Institute of Science and Technology for Brain-Inspired Intelligence, Fudan University; 4 Department of Psychiatry, Shanghai United Family Pudong Hospital, Shanghai, China; 5 Department of Psychiatry, University of Cambridge, Cambridge, United Kingdom

## Abstract

**Introduction:**

Obsessive-compulsive disorder (OCD) is a challenging mental health condition with high treatment-refractory rate, prompting increasing interest in developing non-invasive neuromodulation techniques. Repetitive transcranial magnetic stimulation (rTMS) shows effectiveness in OCD targeting anterior cingulate cortex with a deep H7 coil, but with modest efficacy. The inferior frontal cortex (IFC) plays a critical role in inhibition control, which is a key cognitive domain compromised in OCD. Here, we develop a novel accelerated rTMS protocol employing individualized IFC target combined with symptom provocation techniques.

**Objectives:**

This study aims to comprehensively assess efficacy and safety of our accelerated IFC-rTMS protocol in treatment-refractory OCD.

**Methods:**

Eligible OCD participants (DSM-5) were recruited with the following criteria: age 18 to 60; Yale-Brown Obsessive-Compulsive Scale (Y-BOCS) score≥16; failed pharmacotherapy of at least 2 Selective Serotonin Reuptake Inhibitors (SSRI) or 1 SSRI+1 augmentation. Participants with severe psychiatric or neurological comorbidities were excluded. The study is approved by the Ethics committee of the Shanghai Pudong New Area Mental Health Center.

Participants provided personalized distressing images (rated 4-7 on a 0-10 Visual Analog Scale (VAS)) for a subsequent fMRI task to identify personalized target within IFC, involving passive viewing of neutral and provocation images. RTMS was delivered using a liquid-cooled figure-8 coil. The protocol involved thirty 20-Hz stimulation sequences (1200 pulses in 3 minutes (Miron *et al.* Brain Stimul 2019; 12 1319-1321)), with 6 daily sessions over 5 consecutive days. Stimulation was set at 100% resting motor threshold, preceded by symptom provocation to achieve a VAS distress score of 4-7.

Y-BOCS and Obsessive-Compulsive Inventory-Revised (OCI-R) were assessed at baseline, treatment end and 2 and 4 weeks post-treatment. Treatment response was defined as ≥35% Y-BOCS score reduction.

**Results:**

Eleven participants were recruited and nine finished the treatment. OCD severity was moderate at baseline (Y-BOCS=25.3 ±4.4), with illness duration of 10.7±7.4 years. Both Y-BOCS and OCI-R showed significant differences as a function of treatment (Freidman test χ^2^(3) =11.36, p =0.01; χ^2^(3) =16.36, p<0.001. Fig. 1). The overall response rate at ≥ 1 time point was 66.7%. Adverse events were all mild and self-resolved, including transient headache (n=2), fatigue (n=1) and insomnia (n=1).

**Image 1:**
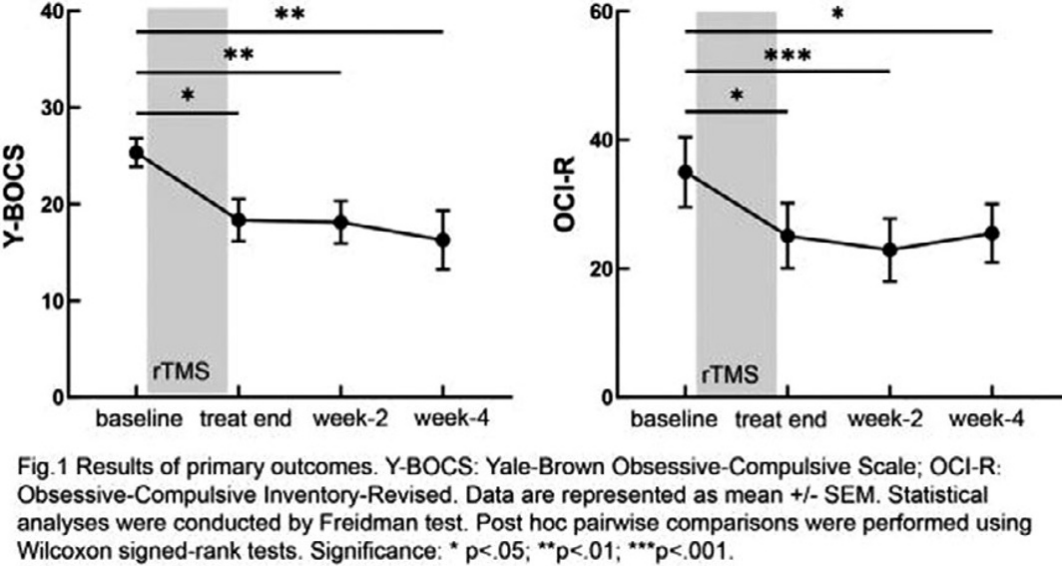
Image 1: Long description.

**Conclusions:**

This proof-of concept case series provides preliminary evidence supporting the feasibility, efficacy and safety of our accelerated IFC-rTMS protocol for treatment-refractory OCD. Further studies with sham-controlled groups are warranted to establish robust clinical evidence and delineate the potential therapeutic mechanisms of this novel approach.

**Disclosure of Interest:**

None Declared

## Long descriptions

### Image 1: Long description

A two-panel figure displaying clinical outcome scores over time.

Left Panel: Y-B O C S graph.

- The vertical Y-axis ranges from 0 to 40.

- The horizontal X-axis lists four time points: baseline, treat end, week-2, and week-4.

- A gray shaded region labeled r T M S covers the period between baseline and treat end.

- The data line starts at a mean of approximately 25 at baseline and drops sharply to roughly 18 at treat end, remaining stable through week-4.

- Horizontal bars at the top indicate statistical significance between baseline and subsequent points: one asterisk for treat end, and two asterisks for week-2 and week-4.

Right Panel: O C I-R graph.

- The vertical Y-axis ranges from 0 to 60.

- The horizontal X-axis follows the same four time points.

- A gray shaded region labeled r T M S is positioned between baseline and treat end.

- The data line starts at a mean of approximately 35 at baseline, decreases to about 25 at treat end, and remains relatively flat through week-4.

- Statistical significance bars at the top show one asterisk for treat end, three asterisks for week-2, and one asterisk for week-4 compared to baseline.

Legend text below the graphs defines Y-B O C S as Yale-Brown Obsessive-Compulsive Scale and O C I-R as Obsessive-Compulsive Inventory-Revised. Data are mean plus or minus S E M. Significance levels are defined as asterisk p < .05, double asterisk p < .01, and triple asterisk p < .001.

Navigate back to Image 1:.

